# The ZIKA Virus Delays Cell Death Through the Anti-Apoptotic Bcl-2 Family Proteins

**DOI:** 10.3390/cells8111338

**Published:** 2019-10-29

**Authors:** Jonathan Turpin, Etienne Frumence, Philippe Desprès, Wildriss Viranaicken, Pascale Krejbich-Trotot

**Affiliations:** PIMIT, Processus Infectieux en Milieu Insulaire Tropical, Université de La Réunion, INSERM UMR 1187, CNRS 9192, IRD 249, Plateforme CYROI, 97490 Sainte-Clotilde, Ile de La Réunion, France; jonathan.turpin@univ-reunion.fr (J.T.); etiennefrum@gmail.com (E.F.); philippe.despres@univ-reunion.fr (P.D.)

**Keywords:** Zika virus, apoptosis, viral replication, Bcl-2 protein family

## Abstract

Zika virus (ZIKV) is an emerging human mosquito-transmitted pathogen of global concern, known to be associated with complications such as congenital defects and neurological disorders in adults. ZIKV infection is associated with induction of cell death. However, previous studies suggest that the virally induced apoptosis occurs at a slower rate compared to the course of viral production. In this present study, we investigated the capacity of ZIKV to delay host cell apoptosis. We provide evidence that ZIKV has the ability to interfere with apoptosis whether it is intrinsically or extrinsically induced. In cells expressing viral replicon-type constructions, we show that this control is achieved through replication. Finally, our work highlights an important role for anti-apoptotic Bcl-2 family protein in the ability of ZIKV to control apoptotic pathways, avoiding premature cell death and thereby promoting virus replication in the host-cell.

## 1. Introduction

Zika virus (ZIKV), which is a flavivirus belonging to the Flaviviridae family, like Dengue virus (DENV), yellow fever virus (YFV) or West Nile virus (WNV), has become a major medical problem worldwide. The human disease known as Zika fever is characterized by mild flu-like symptoms including fever, maculopapular rash, headache and sometimes conjunctivitis, arthralgia and myalgia. Symptoms usually subside within a week [1]. However, during the latest outbreaks, serious pathological features of the disease have been reported. Complications such as microcephaly in newborns or Guillain–Barré syndrome (GBS) in adults were documented during the French Polynesia outbreak in 2013 and in Brazil in 2015 [2,3]. ZIKV is an arbovirus, mainly transmitted to humans through the bite of a mosquito vector from *Aedes* species [4]. Due to an increasingly global distribution of *Aedes*, ZIKV emergence is a threat in many areas that are no longer necessarily located in intertropical areas [5]. The ZIKV particle is composed of a single strand RNA molecule of about 11 kb, inside a nucleocapsid, surrounded by a host-derived membrane that contains two virus encoded proteins (E and M). Phylogenetic analysis of viral sequences has identified two main virus lineages, African and Asian [6], the latter being the main cause of large current epidemics with millions of cases of infection, in particular those that recently affected Brazil and the Americas [7]. Once ZIKV has entered the human body, it targets many types of cells such as epithelial cells, in order to replicate and produce a viral progeny. The life cycle of ZIKV, like other flaviviruses, leads to the release of its single-strand positive sense genomic RNA in the cell cytoplasm where it is translated into a single polyprotein, which is then cleaved by host and viral proteases into three structural proteins (C, prM/M and E), and seven nonstructural proteins (NS; NS1, NS2A, NS2B, NS3, NS4A, NS4B and NS5) [8]. The viral cycle continues with the replication process and the production and maturation of envelope proteins, encapsidation, budding and release of virions by exocytosis.

As with many other viruses, interactions between ZIKV and its host trigger a variety of host responses in the body’s attempt to resolve the infection [9]. Among these responses, apoptosis plays an important role as a host defense mechanism [10]. Apoptosis can quickly remove intracellular niches of viral replication and thus bypass the virus as it multiplies and spreads. As a result, many viruses have developed strategies to evade, delay or divert the cell death responses, often to their advantage [11]. One example is the case of chikungunya virus (CHIKV), an arbovirus of the alphavirus family, which takes advantage of massive apoptosis to hide in disseminating blebs and thus optimizes its spread [12]. Typically, alphaviruses such as CHIKV (but also Sindbis virus, Ross River virus and Semliki Virus) replicate extremely quickly and the infected cells are characterized by rapid and concomitant apoptosis [13]. Unlike alphaviruses, flaviviruses replication is relatively slow. For several of them such as DENV, Japanese encephalitis virus (JEV) and WNV, apoptosis has been shown to be inhibited during the early stages of the viral cycle [14]. The role of viral proteins in the control of apoptosis has been extensively studied, with many observations in support of both pro-apoptotic activity and antiapoptotic effects. For WNV, a nuclear localization of the capsid was shown to induce a caspase-9-dependent apoptosis [15]. Whereas the WNV capsid protein was shown to suppress the activation of caspases 3 and 8 via Akt through a phosphatidylinositol 3-kinase-dependent mechanism (PI3K) [16]. Concerning ZIKV, in cellulo models have shown that infection can lead to cytopathic effects that are typical of apoptosis, and in previous work we observed late-onset apoptosis 48 h after infection of A549 cells with ZIKV isolate PF13 [17]. In some other cell types (human fetal astrocytes), moderate apoptosis can occur even later and possibly contribute to persistent infection [18]. In the particular case of Zika pathology, homeostasis disorder; involving a lack of apoptosis control, a persistent inflammatory response and even viral persistence in the brain has been reported to explain the microcephaly observed in infected newborns [19].

In this study we examined the time course of cellular death associated with ZIKV infection. We confirm that, in A549 cells infected with the epidemic strain from Asian lineage (BeH819015, BR15^MC^), apoptosis is quantitatively moderate and occurs late, after the maximum production of viral progeny. We investigated whether this delay was due to a protective effect of the virus itself. When intrinsic or extrinsic apoptosis was induced within 2 h after infection, we could observe a significant decrease in cell death. As this protection was also obtained in cells expressing ZIKV “replicons”, we deduced that viral replication was efficient at inhibiting apoptosis. ABT-737, an inhibitor of the anti-apoptotic Bcl-2 family proteins, abrogates the protective effects provided by ZIKV. This implies that, with a subversion mechanism that remains to be elucidated, ZIKV is able to maintain an anti-apoptotic status in infected cells while it completes its viral cycle.

## 2. Materials and Methods

### 2.1. Viruses, Cell Lines, Antibodies and Reagents

The clinical isolate PF-25013-18 (PF13) and the molecular clones of ZIKV (BR15^MC^ and MR766^MC^) have been previously described [20,21]. Vero cells (ATCC, CCL-81) and HEK 293T (CRL-3216) were cultured at 37 °C under a 5% CO_2_ atmosphere in MEM medium (PAN Biotech, Aidenbach, Germany), supplemented with 5% or 10% heat-inactivated fetal bovine serum (FBS) (PAN Biotech, Aidenbach, Germany). A549-Dual™ cells (InvivoGen, Toulouse, France, a549d-nfis) designated hereafter as A549 cells were maintained in MEM medium supplemented with 10% heat-inactivated FBS. A549 cells were maintained in growth medium supplemented with 10 μg.mL^−1^ blasticidin and 100 μg.mL^−1^ zeocin (InvivoGen, Toulouse, France).

To detect the ZIKV envelope E protein (ZIKV-E), we used the mouse anti-pan flavivirus envelope E protein mAb 4G2 produced by RD Biotech or the rabbit anti-ZIKV-E-DIII that was kindly provided by Dr. Valerie Choumet (Institut Pasteur). The rabbit anti-BAX (#2772) and anti-active caspase 3 (#9664) antibodies and the mouse anti-cytc 6H2.B4 (#12963) were purchased from Cell Signalling Technology (Ozyme, Saint-Cyr-l’École, France). The mouse anti-BAX clone 2D2 was from Invitrogen (Thermofisher, Les Ulis, France). The anti-mitochondria antibody MTC02-3298 was from Abcam (Cambridge, UK). Donkey anti-mouse Alexa Fluor 488 and anti-rabbit Alexa Fluor 594 IgG antibodies were from Invitrogen (Thermofisher, Les Ulis, France). Horseradish peroxidase-conjugated anti-rabbit (ab97051) and anti-mouse (ab6789) antibodies were purchased from Abcam (Cambridge, UK).

All reagents were from Sigma Aldrich (Humeau, La Chapelle-Sur-Erdre, France) except when indicated. ABT-737 (ab141336) was purchased from Abcam (Cambridge, UK).

### 2.2. Plaque Forming Assay

Viral titers were determined by a standard plaque-forming assay as previously described with minor modifications [17]. Briefly, Vero cells grown in 48-well culture plates were infected with tenfold dilutions of virus samples for 2 h at 37 °C and then incubated with 0.8% carboxymethylcellulose (CMC) for 4 days. The cells were fixed with 3.7% formaldehyde in PBS and stained with 0.5% crystal violet in 20% ethanol. Viral titers were expressed as plaque-forming units per mL (PFU.mL^−1^).

### 2.3. Western Blotting (WB)

Cell lysates were performed in Radioimmunoprecipitation assay buffer (RIPA buffer). All subsequent steps of immunoblotting followed previous descriptions [22,23]. Primary antibodies were used at 1:1000 dilutions. Anti-rabbit immunoglobulin-horseradish peroxidase and anti-mouse immunoglobulin-horseradish peroxidase conjugates were used as secondary antibody (dilution 1:2000). Blots were revealed with enhanced chemiluminescence (ECL) detection reagents using an Amersham Imager 680 (GE, Buc, France).

### 2.4. Induction of Apoptosis

Apoptosis inducers were added 2 h post-infection (hpi) or 2 h before infection (hbi) for ZIKV infected cells. For the replicons, cells were treated 24 h after transfection or passage of stable cells.

For extrinsic apoptosis, cells were treated 2 hpi or 2 hbi with TNFα (10 ng.mL^−1^) and cycloheximide (10 µg.mL^−1^) (TNFα/CHX). The addition of cycloheximide prevents the activation of NFkB and the inhibition of apoptosis [24]. Drugs were added as indicated in the figure legends, before quantification of cell death.

For intrinsic apoptosis, etoposide at 10 µM or blasticidin at 5 µg.mL^−1^ (InvivoGen, Toulouse, France) were added 16 h to 18 h as indicated in the legends. Alternatively, cells were treated with a dose of 400 mJ of UV (Uvitec, Cambridge, UK) and collected for death measurements 16 h after treatment.

For inhibition of the anti-apoptotic Bcl-2 family proteins, ABT-737 (1 µM) was added at the same time as TNF/CHX and the cells were treated as above. Previous experiments were set-up to show that ABT-737 alone under these conditions did not induce cell death (Figure 8B).

### 2.5. Immunofluorescence Assay

A549 and HEK cells grown, infected and treated on glass coverslips were fixed with 3.7% formaldehyde at room temperature for 10 min. Fixed cells were permeabilized with 0.1% Triton X-100 in PBS for 4 min. Coverslips were incubated with primary antibodies (1:1000 dilution) in 1% PBS BSA. Antigen staining was visualized with Alexa Fluor-conjugated secondary antibodies (1:1000, Invitrogen). Nucleus morphology was revealed by DAPI staining. The coverslips were mounted with VECTASHIELD® (Clinisciences, Nanterre, France) and fluorescence was observed using a Nikon Eclipse E2000-U microscope. Images were captured and processed using a Hamamatsu ORCA2 ER camera and the imaging software NIS-Element AR (Nikon, Tokyo, Japan). From the immunofluorescence imaging, percentage of ZIKV-E-positive cells with a stained active mitochondrial BAX or cytosolic cytochrome-c or cleaved caspase 3 was estimated after counting a minimum of six microscopic fields i.e., about 1000 cells examined for each condition.

### 2.6. Cytotoxicity Assay

Necrotic cell damage was evaluated measuring lactate dehydrogenase (LDH) release resulting from a plasma membrane rupture. The supernatant of infected cells was recovered and subjected to a cytotoxicity assay, performed using the CytoTox 96® non-radioactive cytotoxicity assay (Promega, Madison, USA) according to manufacturer’s instructions. Absorbance of converted dye was measured at 490 nm using a microplate reader (Tecan, Grödig, Austria).

### 2.7. Cell Viability Assay (MTT)

MTT (3-[4,5-dimethylthiazol-2-yl]-2,5-diphenyltetrazolium bromide) at 5 mg/mL^−1^ was added on A549 cells cultured in 96-well plate at a density of 5000 cells per well. Following 1 h incubation, MTT medium was removed and the insoluble formazan was solubilized with 100 µL of DMSO. Absorbance of converted dye was measured at 570 nm with a background subtraction at 690 nm.

### 2.8. Caspase-3/7 Activity

Cells were cultured in 96-well plate at a density of 5 × 10^3^ cells per well. Caspase 3/7 activity in crude cell lysates was measured using the Caspase Glo^®^ 3/7 Assay Kit (Promega, Madison, USA) according to the manufacturer’s protocols. Caspase activity was quantified by luminescence using a FLUOstar Omega Microplate Reader (BMG LABTECH, Orthenberg, Germany).

### 2.9. Generation of ZIKV Replicon by the ISA Method

The production of HEK 293T expressing a stable ZIKV RNA replicon with GFP as a reporter protein, named Rep ZIKV-GFP in the study, was based on the sequence of ZIKV strain MR766 Uganda 47-NIID (Genbank access # LC002520) and the ISA (infectious subgenomic amplicons) method as described previously [25]. As a negative control, HEK 293T cells were transfected with pSilencer-puro 2.6 (Ambion, Thermofisher, Les Ulis, France) and pEGFP-C1 (Clontech, Ozyme, Saint-Cyr-l’École, France) plasmids with a ratio of 1 to 10 using lipofectamine 3000 according to supplier’s instructions (Thermofisher, Les Ulis, France) and selected for 5 days in puromycin at 1 µg.mL^−1^.

The production of A549 cells transiently expressing a ZIKV RNA replicon was based on the ISA method and four amplicons overlapping the sequences of BeH819015 isolated in Brazil in 2015 [25] (Figure 7A). Cells were electroporated in the presence of the viral amplicons (Z1 to Z4 fragments) using the Gene pulser II apparatus according to supplier’s instructions (Biorad, Marnes-la-Coquette, France) and treated within 2 days with apoptotic inducers. Cell controls in these experiments were A549DUALtransfected with a plasmid encoding GFP (pEGFP-N1) and A549 transfected with the same amplicons as above but lacking the first segment (the Z1 amplicon) and named REP NEG (Figure 7A).

### 2.10. Statistical Analysis

All values are expressed as mean ± SD of at least three independent experiments, as indicated in figure legends. After normality tests, comparisons between different treatments were analyzed by a one-way ANOVA tests. Values of *p* < 0.05 were considered statistically significant for a post-hoc Tukey’s test. All statistical tests were done using the software Graph-Pad Prism version 7.01. Degrees of significance are indicated in the figure captions as follow: * *p* < 0.05; ** *p* < 0.01; *** *p* < 0.001, **** *p* < 0.0001, ns = not significant.

## 3. Results

### 3.1. ZIKV Does Not Trigger Apoptosis Until the Release of Most of its Progeny

**Figure 1:**
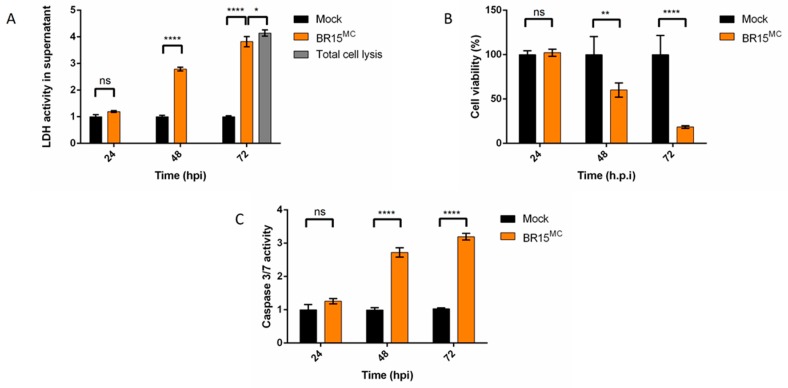
Cell death during a Zika virus (ZIKV) infection of A549 cells. A549 cells were infected with BR15^MC^ at a multiplicity of infection (MOI) of 1. LDH activity was measured in cell supernatant of mock infected cells, BR15 infected cells and in cells treated with triton X-100 as a positive control of total cell lysis value (grey bar) and was normalized to mock infected cells value (**A**), cell viability (MTT assay) (**B**) and caspase 3/7 activity (**C**) were measured at 24, 48 and 72 h post infection (hpi) and normalized to mock infected cells values. Values represent the mean and standard deviation of three independent experiments. Data were analyzed by a one-way ANOVA test with post-hoc Tukey’s test (* *p* < 0.05; ** *p* < 0.01; **** *p* < 0.0001; ns = not significant).

**Figure 2:**
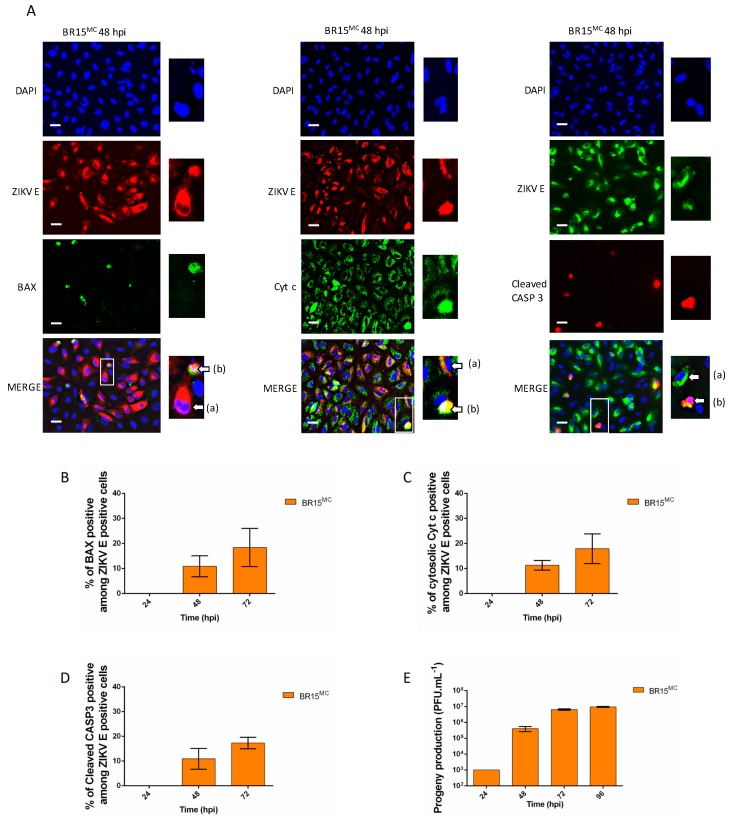
BR15^MC^ does not cause significant activation of apoptosis until late in infection. A549 cells were infected with BR15^MC^ at MOI of 1. (**A**) Cells were immunostained for active mitochondrial BAX, cytochrome c (Cyt c), ZIKV E and cleaved caspase 3 (CASP 3), 48 hpi. The white scale bar represents 10 µm. Right panel series show magnified details of selected cells from the ×200 microscopic field (white square). Arrows indicate (a): an infected cell (stained for ZIKV E) and (b): an infected and apoptotic cell (stained for ZIKV E and with mitochondrial localization of BAX or Cytosolic Cyt c or cleaved CASP3. (**B**) Percentage of A549 infected cells co-immunolabeled for ZIKV E and for active mitochondrial BAX, among the ZIKV E positive cells were determined at 24, 48 and 72 hpi. (**C**) Percentage of A549 infected cells co-immunolabeled for ZIKV E and for cytosolic Cyt c, among the ZIKV E positive cells were determined at 24, 48 and 72 hpi. (**D**) Percentage of A549 infected cells immunostained with anti-cleaved CASP 3 antibody among the ZIKV E positive cells were followed at 24, 48 and 72 hpi. (**E**) The infectious viral particles were collected from infected cell culture supernatants at 24, 48, 72 and 96 hpi and titrated. Values represent the mean and standard deviation of three independent experiments.

Our research team had previously demonstrated that a South Pacific epidemic clinical isolate of ZIKV (PF13-25013-18) was able to infect A549 epithelial cells. These cells are particularly permissive to the virus and therefore constitute a suitable model for studying in cellulo host-virus interactions [17]. In order to characterize the cellular death profile that accompanies ZIKV infection more precisely, we conducted a study of the cytopathic effects induced with the viral molecular clone of the epidemic strain from Asian lineage, BeH819015 isolated in Brazil in 2015 (BR15^MC^) [21]. We infected A549 cells with BR15^MC^ at a multiplicity of infection (MOI) of 1 and followed for 3 days, the characteristics of the viro-induced cell death (Figure 1). We further monitored the induction and execution of apoptosis specifically in infected cells to compare them with the results of viral production (Figure 2).

**Figure 3:**
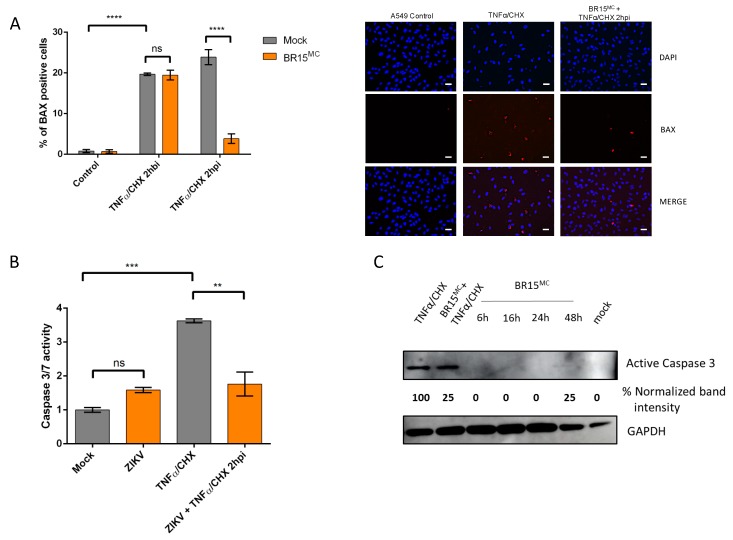
BR15^MC^ provides a protection against extrinsically induced cell death. A549 cells were infected with BR15^MC^ at MOI of 1 for 8 h and treated with TNFα and cycloheximide (TNFα/CHX) 2 h post infection (2 hpi) or 2 h before infection (2 hbi). (**A**) The percentage of A549 cells immunolabeled with anti-BAX antibody was enumerated from images representative of immunofluorescence observations (panels on the right, white scale bar: 10µm). (**B**) Caspase 3/7 activity was measured after TNFα/CHX treatment 2 hpi and normalized to mock treated and infected cells. (**C**) Immunoblot of active caspase 3 during TNFα treatment 2 hpi and infection time course with BR15^MC^. Active caspase 3 band intensity was normalized with GAPDH. Western blotting (WB) is representative of three independent experiments. Values represent the mean and standard deviation of three independent experiments. Data were analyzed by a one-way ANOVA test with post-hoc Tukey’s test (** *p* < 0.01; *** *p* < 0.001, **** *p* < 0.0001, ns = not significant).

The measurement of LDH activity in infected cell culture supernatants, which results from a loss of cell integrity mainly reflecting secondary necrosis or estimation of cell viability by measurement of mitochondrial activity by MTT assay, revealed that cell mortality was detected at 48 h post infection (hpi) to reach a high level 72 hpi (Figure 1A,B). At 24 hpi there was no detectable sign of cell death by apoptosis when we looked at the activity or presence of cleaved caspase 3 (Figure 1C and Figure 2C). Relocalization of the pro-apoptotic factor BAX to mitochondria, an early marker of apoptosis (Supplemental Figure S1A), was only observed at 48 hpi (Figure 2A,B) and only occurred in approximately 10% of the cells that were immunolabeled with an antibody directed against the viral envelope protein E (ZIKV-E; Figure 2B). Examination of another signal of engagement in apoptosis, namely the presence of cytosolic cytochrome-c (Figure 2A, magnified image with (b) arrow on the cyt-c immunodetection panel), led to the same observation (Figure 2C). It should be noted that only some of the cells immunolabeled for ZIKV-E had apoptotic characteristics (Figure 2A). The percentage of uninfected cells with signs of death by apoptosis was always equivalent to that observed in the controlled cell cultures over time (Figure S1B). Analysis of apoptosis execution, such as the measurement of caspase 3/7 activity (Figure 1C), immuno-detection in infected cells of cleaved activated caspase 3 (Figure 2A,D) or by Western blotting (WB; Figure 3C) supported the delay in cell death with respect to the course of viral multiplication. Moreover, significant signs of engagement in apoptosis occurred when the released viral progeny have already reached their maximum (Figure 2E). A late and low proportion of ZIKV-infected cells engaged in apoptosis were also retrieved during the infection of Vero cells (Figure S2). This apoptosis kinetics differed significantly from the one induced during the Ross River alphavirus (RRV) infection. Thus during RRV infection the maximum of caspase 3 activity was observed at 24 hpi and was at least 10 fold higher than the one measured in ZIKV-infected cells. These observations suggest a ZIKV-infection specific feature of the viro-induced apoptosis, together with a regulatory mechanism implemented by the ZIKV to delay apoptosis.

To rule out that delayed apoptosis in infected cells was not a feature of the epithelial cell line A549 or Vero cells, we verified the respective courses of infection and cell death in other cell models. In the U251MG line of human brain glioblastoma-astrocytoma cells, infection kinetics was accompanied by a complete absence of apoptosis within the first 48 h of infection before the maximum of viral progeny production (Figure S3). Thus we confirm that death by apoptosis induced by our BR15^MC^ viral molecular clone as for the Asian epidemic clinical isolate PF13 occurs late and is relatively moderate compared to the kinetics of induced viral death that can be observed in the case of infection by other arboviruses like alphaviruses (Figure S2 and [12]).

### 3.2. ZIKV Infected Cells Display Resistance to Apoptosis Inducers

Apoptosis can be initiated by extrinsic or intrinsic pathways, the former being mediated by death receptors located on the cell surface, the latter being driven by various cellular stresses such as DNA damage. Activation of apoptosis-initiating caspases 8 or 9 results in mitochondrial outer membrane permeabilization (MOMP) via oligomerization and insertion of the proapoptotic factors BAX/BAK in the mitochondria of type II cells such as epithelial cells [26].

**Figure 4:**
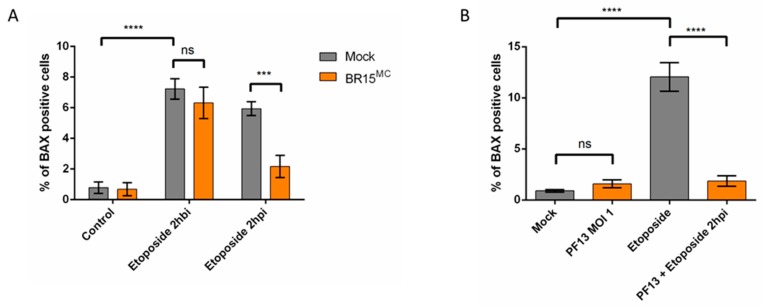
ZIKV provides a protection against intrinsically induced cell death by etoposide. (**A**) A549 cells were infected with BR15^MC^ at MOI of 1 for 8 h and treated with etoposide 2 h before infection (2 hbi) or 2 h post infection (2 hpi). The percentage of A549 cells immunostained with anti-BAX antibody was followed. (**B**) A549 cells were infected with PF13 at MOI of 1 for 8 h and treated with etoposide for 16 h, 2 hpi. The percentage of A549 cells immunostained with anti-BAX antibody was followed. Values represent the mean and standard deviation of three independent experiments Data were analyzed by a one-way ANOVA test with post-hoc Tukey’s test (*** *p* < 0.001, **** *p* < 0.0001, ns = not significant).

The late onset of apoptosis in infected cells led us to postulate that ZIKV may modulate the apoptotic response of the cell, delaying it through transient inhibition. To test this hypothesis, we investigated whether ZIKV could counteract the effect of death inducers added during the infection time course. We induced apoptosis through extrinsic and intrinsic pathways and tested the addition of the inducer at 2 h prior-to and 2 h post infection (Figure 3 and Figure 4).

#### 3.2.1. ZIKV Provides Protection Against Death Receptor Mediated Cell Death

To drive an extrinsic apoptosis, we induced the TNF-receptor using its ligand, TNF-alpha (TNFα), inhibiting the cytoprotective NFkb response by the addition of the translation inhibitor cycloheximide (CHX). Between 6 and 8 h of treatment leads to an estimated 20% cell mortality in A549 cells, when counting BAX positive cells (Figure 3A). No variation in the percentage of cells engaged in early apoptosis (BAX+) 8 h after the onset of treatment was observed when TNFα/CHX was added 2 h prior to ZIKV infection.

Conversely, if TNFα/CHX was added 2 h post infection, a drastic and significant drop in the percentage of cells engaged in apoptosis was observed. This drop was corroborated by the measure of caspase 3 activity (Figure 3B). It is worth recalling that in this lapse of time, virally induced apoptosis is undetectable and therefore is unlikely to interfere with the quantification of cell death induced by the action of TNFα/CHX. Moreover, in Western blots, detectable levels of the active form of caspase 3 in BR15^MC^-infected cells were seen to be reduced by around 75% after treatment with TNFα/CHX (Figure 3C).

#### 3.2.2. ZIKV Provides Protection Against Intrinsically Induced Cell Death

Intrinsically induced apoptosis was stimulated by chemical DNA damage. To do this, we used the genotoxic agent etoposide, a topoisomerase-II inhibitor, which causes chromosome breaks during DNA replication. Overnight treatment with etoposide (16–18 h) resulted in a percentage of cells with a mitochondrial BAX among the remaining adherent cells that was between 7% and 12%, depending on the experiment (Figure 4). Similar to the induction of cell death by TNFα/CHX, although the effects are slightly more modest, apoptosis produced in A549 cells after 16 h of etoposide treatment was significantly reduced in the case where ZIKV was added 2 h post-infection (Figure 4A). In addition, both BR15, the molecular clone of ZIKV (Figure 4A) and a clinical isolate of the epidemic strain PF13 (Figure 4B) were capable to interfere with apoptosis induction by etoposide.

**Figure 5:**
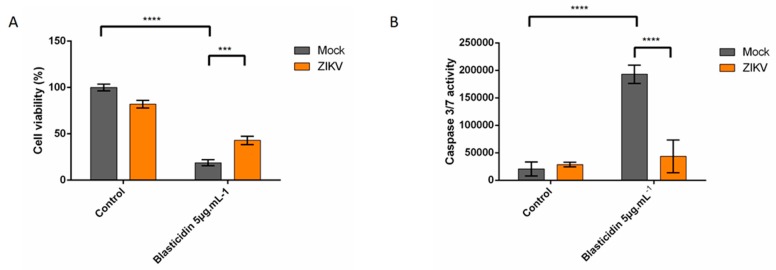
ZIKV provides a protection against intrinsically induced cell death by blasticidin. Vero cells were infected with ZIKV-PF13 at MOI of 1 for 8 h and treated with blasticidin for 16 h, followed by viability assay (MTT) (**A**) or caspase 3/7 activity (**B**). Values represent the mean and standard deviation of three independent experiments. Values represent the mean and standard deviation of three independent experiments Data were analyzed by a one-way ANOVA test with post-hoc Tukey’s test (** *p* < 0.01; *** *p* < 0.001, **** *p* < 0.0001, ns = not significant).

We also tested the effect of the cell line used for infection by repeating the procedure in Vero cells, in this instance using blasticidin to induce intrinsic apoptosis in response to the inhibition of translation instead of DNA damage with etoposide. Following cell death by MTT assay and caspase 3/7 activity again suggested that ZIKV infection represses apoptosis, regardless of cell type and death inducers (Figure 5A,B).

In order to ensure that the ZIKV mediated repression of apoptosis was conserved between ZIKV strains, we looked at the effect of apoptosis induction with etoposide after infection with the epidemic clinical isolate PF13 (ZIKV-PF13) but also with a molecular clone of historical strain from African lineage, MR766-NIID isolated in Uganda in 1947 (MR766^MC^; Supplemental Figure S4). ZIKV infection resulted in reduced apoptosis for all tested strains. Convergent measurements of several parameters that establish the death rate and degree of protection confirm that ZIKV interferes with the achievement of apoptosis in response to a death signal.

### 3.3. Apoptosis is Repressed Through ZIKV Replication

Our data suggest that protection against an exogenous induced apoptosis is acquired once the virus has entered the cells and started its multiplication cycle. In order to determine the contribution of the replicative process in the protective mechanism, we exploited the “replicon” systems.

#### 3.3.1. Cells Stably Expressing a ZIKV Replicon are Protected from Intrinsically and Extrinsically Mediated Apoptosis

**Figure 6:**
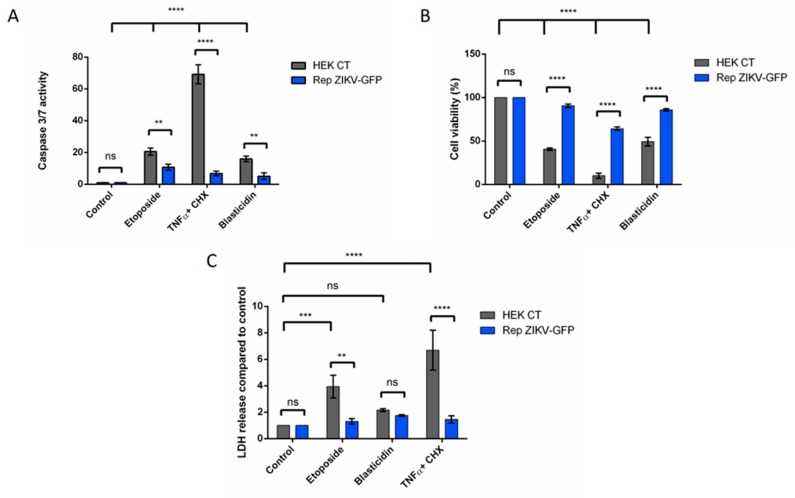
ZIKV replicon-expressing cells are protected from apoptosis. HEK-293 cells stably expressing a ZIKV replicon (Rep ZIKV-GFP) are protected from intrinsically and extrinsically mediated apoptosis. HEK-293 cells with Rep ZIKV-GFP were treated with TNFα and CHX for 6 h, or treated with etoposide 10 µM or blasticidin 25 µg/mL^−1^ for 16 h and analyzed for: caspase 3/7 activity (**A**), cell viability (MTT assay) (**B**) and released LDH activity (**C**). Values represent the mean and standard deviation obtained with three different clones of Rep ZIKV-GFP. Values represent the mean and standard deviation of three independent experiments. Data were analyzed by a one-way ANOVA test with post-hoc Tukey’s test (** *p* < 0.01; *** *p* < 0.001, **** *p* < 0.0001, ns = not significant).

Since both epidemic Asian (BR15^MC^) and historical African (MR766^MC^) strains of ZIKV were found to be able to control apoptosis, we investigated the role of viral replication in the mechanism of apoptosis repression using a previously established MR766 replicon system in HEK-293 cells [25]. The Rep ZIKV-GFP cells have a self-replicating RNA encoding the viral NS proteins from MR766-NIID, a puromycin resistance gene to facilitate selection and GFP as a reporter. HEK-293 cells stably transfected with a plasmid encoding a GFP reporter gene and puromycin resistance without any viral material was used as a control (HEK CT; Figure 6). Apoptosis was induced either with TNFα/CHX, with etoposide or with blasticidin. We monitored apoptosis and in particular measured caspase 3/7 activity 6h post addition of TNFα/CHX or 16 h post addition of etoposide or blasticidin. We compared the values related to caspase 3/7 activity in Rep ZIKV-GFP with those obtained with the control cells (HEK CT; Figure 6A).

Monitoring apoptosis by caspase 3/7 activity (Figure 6A), together with the measure of cell viability (Figure 6B) and released LDH activity (Figure 6C) demonstrated that ZIKV replicon resulted in a significant reduction in the indicators of cell death. An inhibition of apoptosis was not observed in the case of the cell control, selected for GFP and puromycin resistance (HEK CT) as well as in untransfected HEK-293 cells (data not shown). It can be concluded that resistance to puromycin or the presence of a GFP encoding gene were not responsible for a protective effect.

It can therefore be proposed that the presence of an autonomous replication of a viral RNA associated with the expression of ZIKV NS proteins makes cells resistant to several extrinsic and intrinsic apoptotic inducers.

#### 3.3.2. A549 Cells Transiently Expressing a ZIKV Replicon are Protected from Different Intrinsically Induced Apoptosis

**Figure 7:**
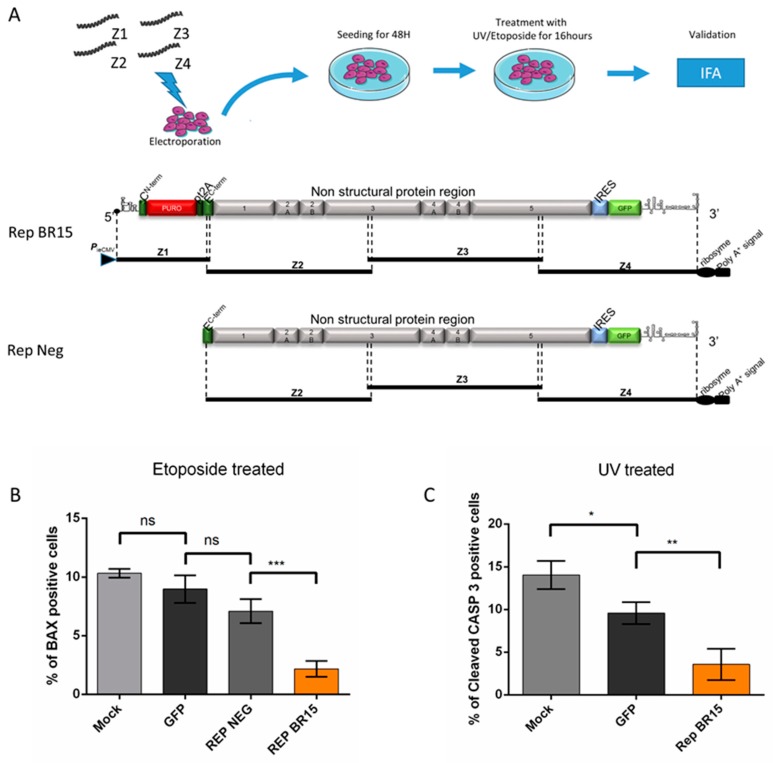
A549 cells transiently expressing a ZIKV replicon are protected against cell death by apoptosis. A549 cells were transfected with ZIKV amplicons (Z genomic overlapping fragments) for production of REP BR15 (Z1, 2, 3 and 4) and REP NEG (Z2, 3 and 4) or with pEGFP-N1 (**A**). At 48 h after transfection A549 cells were treated with etoposide for 16 h (**B**) or UV at 400 mJ (**C**). The percentage of A549 cells immunostained with anti-BAX antibody or anti-cleaved CASP 3 was monitored. Values represent the mean and standard deviation of three independent experiments. Data were analyzed by a one-way ANOVA test with post-hoc Tukey’s test (* *p* < 0.05; ** *p* < 0.01; *** *p* < 0.001, ns = not significant).

To address a protective effect of the viral replication in a system consistent with the one in which the effect was revealed through infection, we adapted the ISA method to obtain A549 cells transiently expressing a ZIKV replicon with the non-structural sequences from BeH819015 (REP BR15). We used A549 cells transfected with an incomplete set of amplicons as a negative control (REP-NEG; Figure 7A). Apoptosis was induced 48 h after amplicon transfection either by etoposide treatment (Figure 7B) or by DNA damage through exposure to UV light (Figure 7C).

In cells treated with etoposide, REP BR15 expressing cells showed approximately half the number of apoptotic cells of the replicon control (REP NEG; Figure 7B). The percentage of dying cells was even lower in cells expressing REP BR15 after UV treatment (Figure 7C). Thus, REP BR15 was able to confer resistance to apoptosis. This resistance was also observed in an experiment conducted with A549 cells transiently expressing a ZIKV replicon from the MR766 strain of ZIKV (Figure S5). All together these results suggest that replication of the ZIKV was capable of inhibiting apoptosis.

### 3.4. ZIKV Promotes an Anti-Apoptotic Prevailing Status in Infected Cells Through the Bcl-2 Family Protein

**Figure 8:**
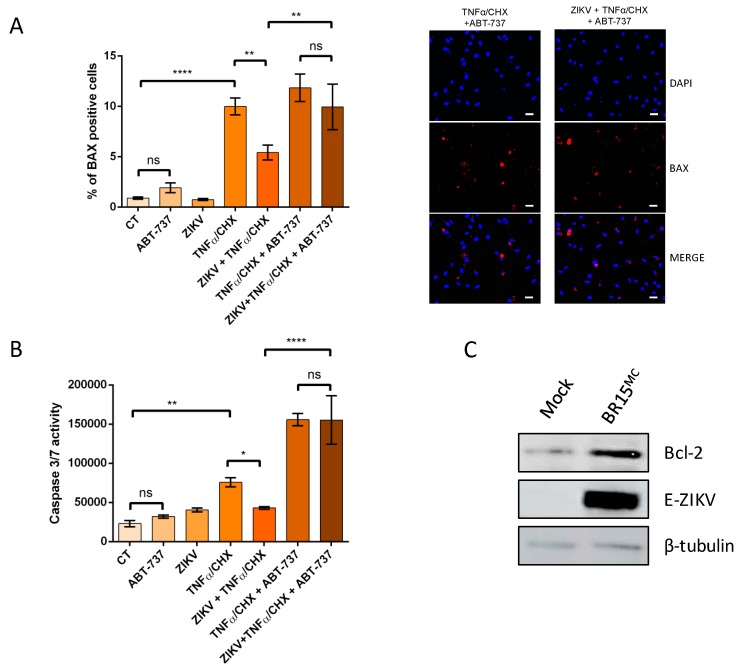
Inhibition of anti-apoptotic Bcl-2 family proteins abrogates the protection mediated by ZIKV. A549 cells were infected with ZIKV at MOI of 1. TNFα and CHX were added 2 h post infection for 6 h with or without ABT-737. A549 cells were immunostained with an anti-BAX antibody (representative images in panels on the right, white scale bar: 10µm) (**A**) and caspase 3/7 activity was followed after treatment (**B**). A549 cells were infected with ZIKV at MOI of 1 and level of Bcl-2 was followed by western blot 24 h post infection (**C**). Values represent the mean and standard deviation of three independent experiments. Data were analyzed by a one-way ANOVA test with post-hoc Tukey’s test (* *p* < 0.05; ** *p* < 0.01; *** *p* < 0.001, **** *p* < 0.0001, ns = not significant).

The long delay in the onset of viral apoptosis induced by ZIKV infection, particularly in the case of Asian viral strains responsible for current epidemics, and the demonstration that, when the viral RNA is present and replicating there is an inhibition of apoptotic induction, suggest that cells have acquired with the virus a status in which anti-apoptotic activity prevails. The protective effect acquired with ZIKV could be at the level of convergence of the intrinsic and extrinsic pathways. As we followed BAX relocalization we could argue in favor of protection around the mitochondria events and the control of the outer membrane permeabilization (OMP). A prominent anti-apoptotic factor involved in the regulation of early apoptosis, by operating mainly at the mitochondrial level for the control of its permeabilization is Bcl-2 and the related Bcl-XL protein [27]. To identify to which extent these anti-apoptotic factors play a role in the protection provided by ZIKV, we examined the effect of ABT-737 on cell death outcomes, with or without BR15^MC^ (Figure 8A,B). ABT-737 is a BH3 mimetic molecule that can bind to the hydrophobic groove of the members of the anti-apoptotic Bcl-2 protein family, Bcl-2 and Bcl-xL, and therefore inhibits their activity by shifting oligomerization mechanisms in favor of BAX/BAK dimerization [28].

When inducing apoptosis with TNFα/CHX, addition of ABT-737 restored the percentage of ZIKV-infected cells with mitochondrial BAX (Figure 8A) and caspase 3/7 activity (Figure 8B) to levels that were similar to cells that were not infected with ZIKV.

These observations suggest that ABT-737 has counteracted the protective effect acquired with the virus. It can legitimately be deduced that the viro-induced protective effect probably depends on the anti-apoptotic activity of Bcl-2 family proteins. When Bcl-2/Bcl-XL is inhibited, ZIKV no longer allows a quantitative reduction of apoptosis.

As we used cycloheximide or blasticidin in our assays, we could therefore assume that the mechanism implemented by ZIKV did not involve a “de novo” synthesis of proteins. Based on these remarks, we formulated the hypothesis that ZIKV might allow stabilization of the Bcl-2 protein over time. We performed a western blot assay to measure Bcl-2 protein level upon ZIKV infection. Upon ZIKV infection, we observed an increase of Bcl-2 at the protein level (Figure 8C). This stabilization of Bcl-2 could support the anti-apoptotic capacity of ZIKV.

## 4. Discussion

ZIKV has attracted tremendous attention in the field of medically important flaviviruses, which are responsible of world epidemics that are difficult to control [29]. To date, there is no effective treatment against this emerging pathogen despite significant efforts made to succeed in providing an effective vaccine. The characterization of the interaction modalities between the pathogen and host cells are important in order to better understand the strategies adopted by the virus to be effective in its replication and dispersal of its progeny. Among the responses of infected cells, a high priority must be given to virally induced apoptosis since its completion can significantly interfere with the virus’s multiplication cycle and hinder its spread [30]. Moreover, crosstalk between innate immune signaling and cell death pathways and how the viruses are able to manipulate each other are essential for viral clearance or persistence and for the global outcomes of the infection. Many studies support that ZIKV infection induces apoptosis in vivo as well as in vitro [31,32,33] However, several of them also assert that ZIKV induced cell death could be delayed [17,18]. We therefore wanted to define whether this capacity was specific to the strain of ZIKV responsible for the current epidemic and whether ZIKV was able to interfere with the induction of apoptosis.

Using a molecular clone of the BeH819015 from the Brazilian 2015 outbreak (BR15^MC^) and its comparison to the clinical isolate from the French Polynesian 2013 outbreak (PF13), our work has highlighted that ZIKV strains from the actual epidemic Asian lineage are particularly inclined to delay the onset of apoptosis in infected human epithelial cells (A549) as well as in human brain glioblastoma–astrocytoma cells (U251 MG; Figure 1, Figure 2 and Figure S3). These viruses are also characterized by a rather slow viral growth compared to the molecular clone of the historical strain of ZIKV MR766 from the African lineage [21]. Infection with MR766^MC^ was marked by a higher cytotoxicity over the duration of infection, with 10% of infected cells showing signs of entry into apoptosis as early as 24 h, whereas epidemic strains had no signs of mortality before 48 h. However, despite this behavior of MR766^MC^, which seems more aggressive, it must be admitted that the mortality rate among infected cells remained limited until 48 h post infection (Figure 2 and Figure S4). We proposed from these results that both viruses had the ability to interfere with the onset of apoptosis even though faster and more effective viral growth in the early hours of infection for MR766^MC^ than for BR15^MC^ had resulted in faster and more cytopathic effects. In both cases, the maximum mortality was only recorded when the virion production had reached its highest titer. This observation suggests a manipulation of apoptosis orchestrated by ZIKV in order to give it enough time to complete its entire production cycle. This phenomenon has already been described for other flaviviruses such as DENV, JEV and WNV as they can delay apoptosis through activation of the phosphatidylinositol 3-kinase (PI3K) and Akt pathway [16,34].

In a rather unexpected way, our work mainly shows that in the early stages of infection, ZIKV infection provides a solid protection against an exogenous induced cell death. We provided supporting evidence that protection is acquired both with the Asian epidemic strains and with the African strain (Figure 3, Figure 4, Figures S4 and S5). This protection is effective against apoptosis mediated by an extrinsic death inducer (TNFα) as well as by an intrinsic signal (provided by the action of etoposide or blasticidin). Resistance to these induction modes has also been found to characterize cells expressing replicons, either HEK 293 cells stably expressing a MR766 replicon or A549 cells transiently expressing BR15 replicons or MR766 replicons (Figure 6, Figure 7 and Figure S5). The data obtained with the use of these replicons are in support of a greater protection granted by BR15. This is consistent with the data obtained with the whole virus that is responsible for the longest delay in apoptosis entry. Cell death inhibition ability acquired with the ZIKV replicons would imply that the single presence of a viral RNA leading to the production of the NS proteins and allowing its self-replication is the driving force behind the protection acquired against apoptosis.

**Figure 9:**
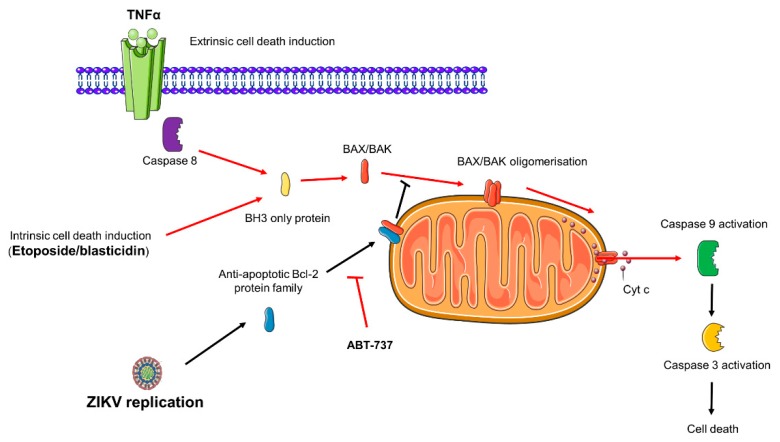
Model depicting the protective action of ZIKV infection against apoptosis. Intrinsic or extrinsic activation of cell death occurs through the formation of mitochondrial outer membrane pore (MOMP) via the BAX/BAK complex. The anti-apoptotic family Bcl-2 members Bcl-2/Bcl-XL interfere with the complex formation by sequestering BAX via their BH3 domain. ZIKV replication interferes with BAX relocalization at the mitochondria. ABT-737, a BH3 mimetic, which inhibits Bcl-2/Bcl-XL, abrogates the inhibition induced by ZIKV. Indeed, the virus delays apoptosis during infection by modulating the homeostasis of Bcl-2/Bcl-XL.

This discovery is rather unusual when one considers the literature mainly in favor of pro apoptotic functions for the NS proteins [35]. However, studies have also shown that some NS may help ZIKV to evade antiviral immunity and cell death. NS2B in particular was proposed to be responsible for blocking (RIG-I)-like receptors triggered apoptotic cell death [36]. A thorough identification of the viral protein responsible for an inhibitory effect needs to be confirmed and further investigated. While our work suggests a possible role for NS proteins in the control of apoptosis, we cannot rule out the possibility that structural proteins may also act. Anti-apoptotic activities have been previously reported in relation to the capsid [16,18]. It should be noted that in the construct we used to generate the replicons, the polyprotein produced retains the first 33 amino acids of the capsid. It would be interesting to see if this part of the structural protein, released after the cleavage of the GFP has a role in protection. We could not also exclude a role of the viral RNA by itself. Recent work has also investigated the effects of viral RNAs, as flaviviruses are known to produce multiple small RNAs that may have interference activities in the cell physiology. It was recently described that recent epidemic Asian lineage display more negative-strand replicative intermediates than the historical African strain [37]. This important production could be a key element in the search for which viral determinants are crucial in the control of apoptosis. We also know how important are the viral genomic 3′ UTR regions and the Subgenomic Flavivirus RNA (sfRNA) produced, in the implementation of cellular responses to infection [38]. It remains to be discovered which factor associated with the replication process of ZIKV viral RNA is involved in the protection mechanism. What we already know from our study is that this mechanism required the Bcl-2 family protein as ABT-737 abrogates the protection acquired against apoptosis by ZIKV infection (Figure 8). The anti-apoptotic capacity of the pro-survival Bcl-2 proteins is known to depend mainly on their ability to sequester pro-apoptotic proteins by binding their BH3 domains. A decrease in Bcl-2 leads to the disruption of associated pro-survival and pro-apoptotic Bcl-2 proteins and will promote apoptosis whereas overexpression of Bcl-2 will inhibit mitochondrial OMP [39,40]. A control of the stability and degradation of Bcl-2 is therefore a key in the subtle balance that takes place between pro and anti-apoptotic suits to determine the cell’s fate [40]. A previous study showed the importance of Bcl-xL for cells survival, in deficient Bcl-2 cells during ZIKV infection [41], but here we could not exclude a role for Bcl-2 and/or Bcl-xL protein as our model expressed these two anti-apoptotic proteins. Based on the fact that Bcl-2 family protein was involved in ZIKV infection with a Bcl-2 protein level quantitatively maintained (Figure 8), we formulated the hypothesis that ZIKV might allow a stabilization of the Bcl-2 protein over time and inhibit MOMP formation (Figure 9). The mechanism by which the virus allows the Bcl-2 stabilization and blocks apoptosis needs further investigation.

## Supplementary Materials

The following are available online at https://www.mdpi.com/2073-4409/8/11/1338/s1, Figure S1. IF imaging to validate markers of apoptosis in A549 cells. Figure S2. Alphavirus RRV induces early and massive apoptosis compared to Zika virus. Figure S3. ZIKV-PF13 does not cause significant activation of apoptosis until late in infection in U251MG cells. Figure S4. ZIKV-MR766 does not cause significant activation of apoptosis until late in infection and ZIKV-MR766 is able to control cell death. Figure S5. A549 cells transiently expressing a ZIKV-MR766 replicon are protected against cell death by apoptosis.
