# Supplementary material for: The ZIKA Virus Delays Cell Death Through the Anti-Apoptotic Bcl-2 Family Proteins
Source: Cells. 2019 Oct 29;8(11):1338. doi: 10.3390/cells8111338 (PMC6912272; doi:10.3390/cells8111338)
Supplement: Supplementary file 1 [file cells-08-01338-s001.pdf]

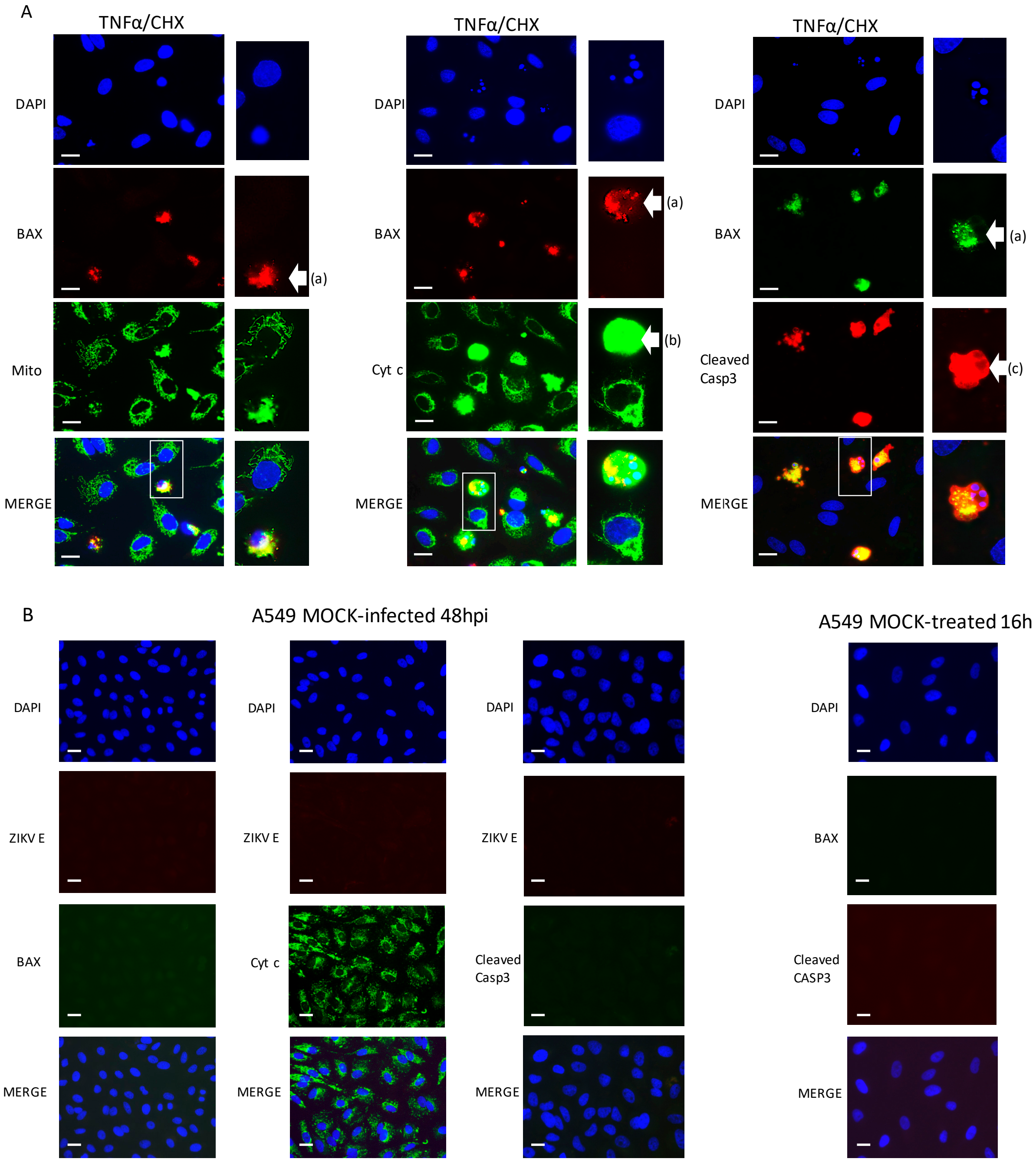
A
TNFα/CHX
TNFα/CHX
TNFα/CHX
DAPI
BAX
Mito
MERGE
Cyt c
Cleaved
Casp3
(a)
(b)
(c)
B
A549 MOCK-infected 48hpi
A549 MOCK-treated 16h
ZIKV E
Cleaved
CASP3

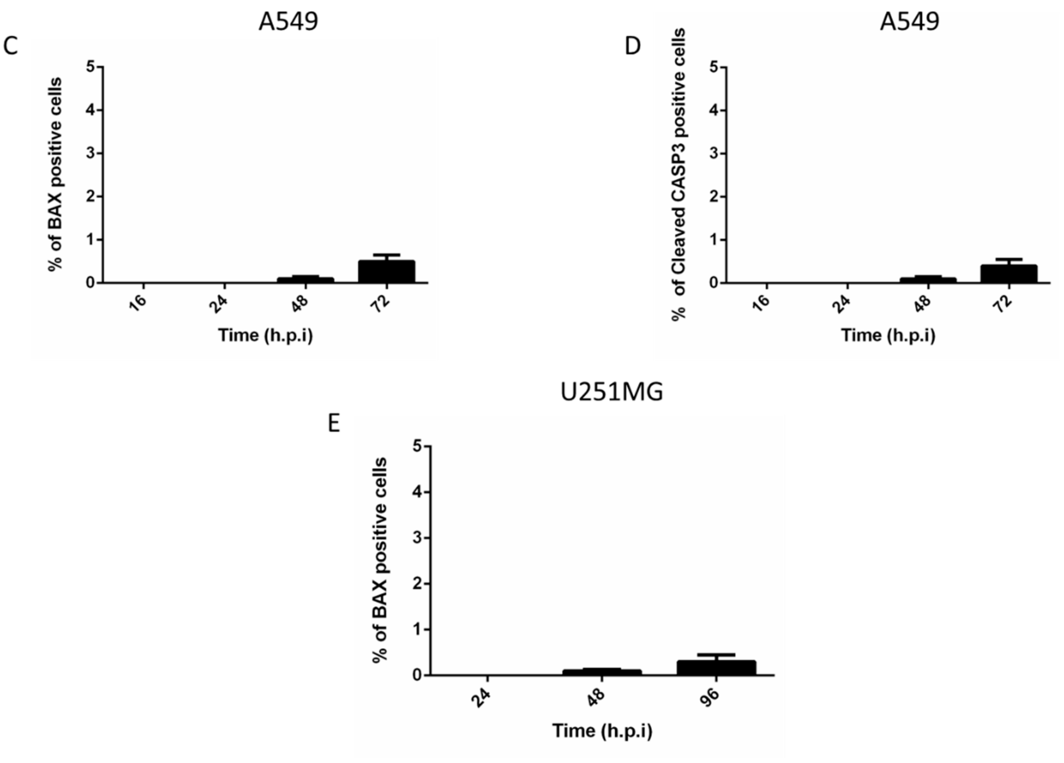

Figure 1. IF imaging to validate markers of apoptosis in A549 cells. (**A**) A549 cells were treated with TNF/CHX for 16h and co-immunolabeled with anti-BAX Ab and anti-mitochondria Ab (mito) (left panel), anti-cytochrome c Ab (cyt c) (middle panel), anti-Cleaved Caspase 3 (Cleaved CASP3) (right panel). The white scale bar represents 10µm. Magnified details of selected cells from the x200 microscopic field (white square) are displayed next to the images. Arrows indicate apoptotic characteristics (a): BAX mitochondrial staining, (b): cytosolic cytochrome c staining, (c) cleaved CASP3 staining. (**B**) A549 cells were mock-infected for 48 hours and cells were immuno-labelled with anti-E and anti-BAX Ab (left panel), anti-cytochrome c Ab (cyt c) (middle panel) or anti-Cleaved Caspase 3 Ab (Cleaved CASP3) (right panel). Mock-treated A549 for 16 h were immuno-labelled with anti-BAX Ab and anti-Cleaved Caspase 3 Ab (white scale bar: 10µm). Percentage of A549 cells with mitochondrial staining of BAX (**C**) or with a Cleaved Caspase 3 staining (**D**) and percentage of U251MG with mitochondrial staining of BAX (**E**) during the time course experiments. Values represent the mean and standard deviation of three independent experiments.

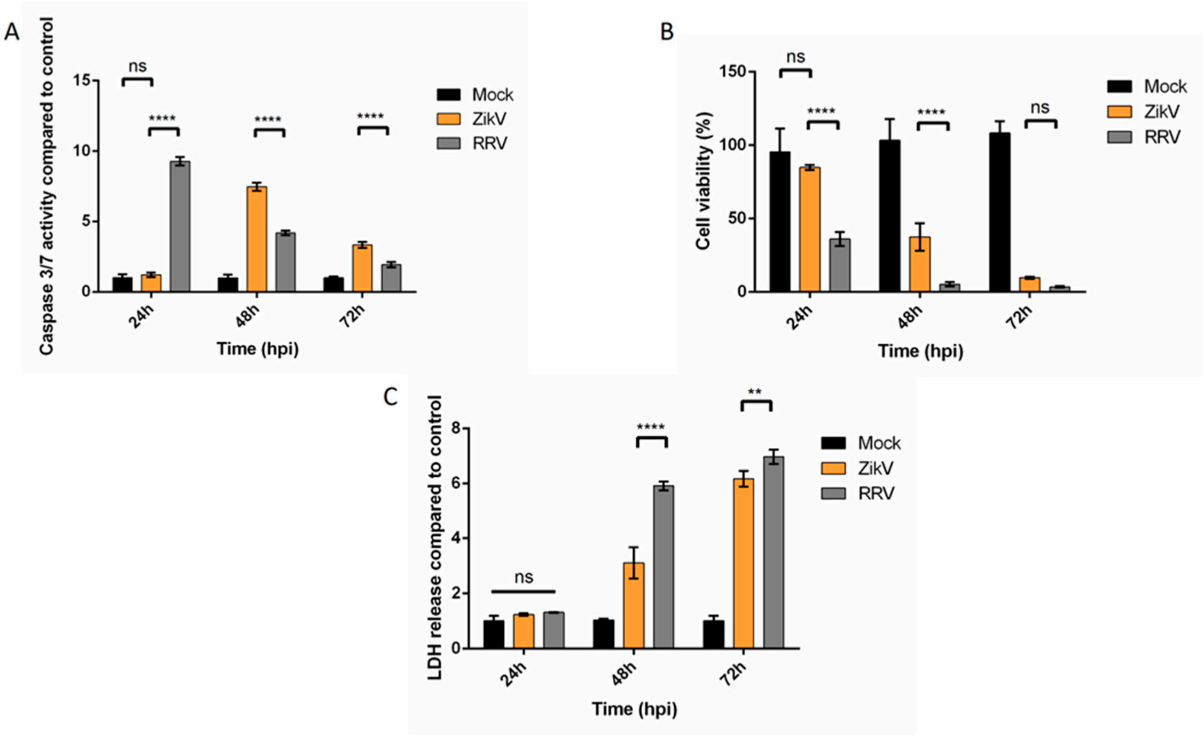

Figure 2. Alphavirus RRV induces early and massive apoptosis compared to Zika virus. Vero cells were infected with Ross River virus (RRV) or with ZIKV at an MOI of 1 for 72 h and analysed for (**A**) caspase 3/7 activity, (**B**) cell viability assay (MTT) and (**C**) released LDH activity in cell supernatants. Values represent the mean and SD of three independent experiments. Data were analyzed by a one-way ANOVA test with post-hoc Tukey's test. (**p<0.01; ***p<0.001, ****p<0.0001, ns = not significant).

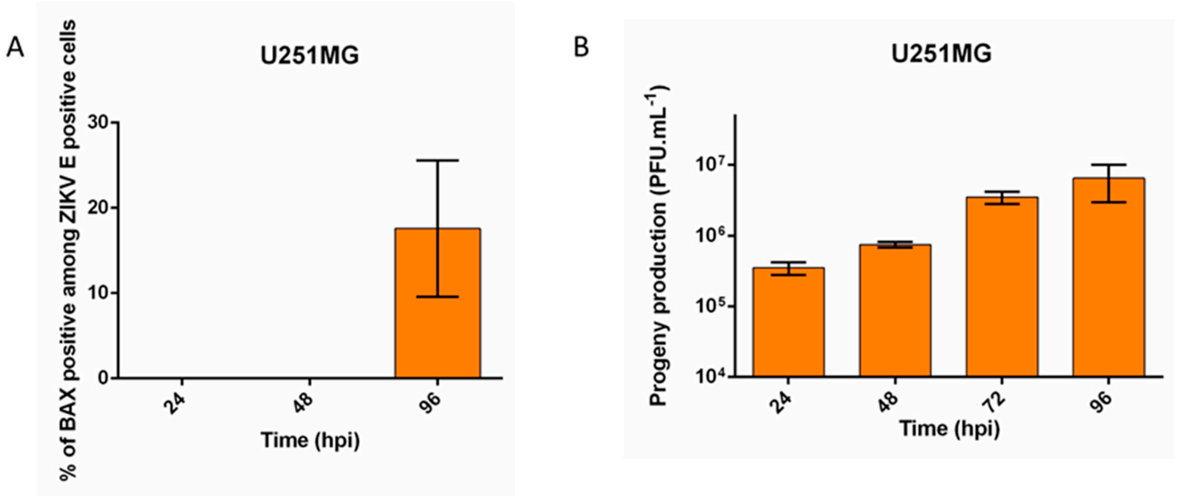

Figure 3. ZIKV-PF13 does not cause significant activation of apoptosis until late in infection in U251MG cells. U251MG cells were infected with ZIKV PF13 (clinical isolate) at MOI of 1 for 96h. (**A**) Percentage of U251MG cells infected immunostained with anti-BAX antibody were determined at 24, 48 and 96 hpi. (**B**) The infectious viral particles were collected from infected cell culture supernatants during 96 h post infection and titrated. Values represent mean of three independent experiments.

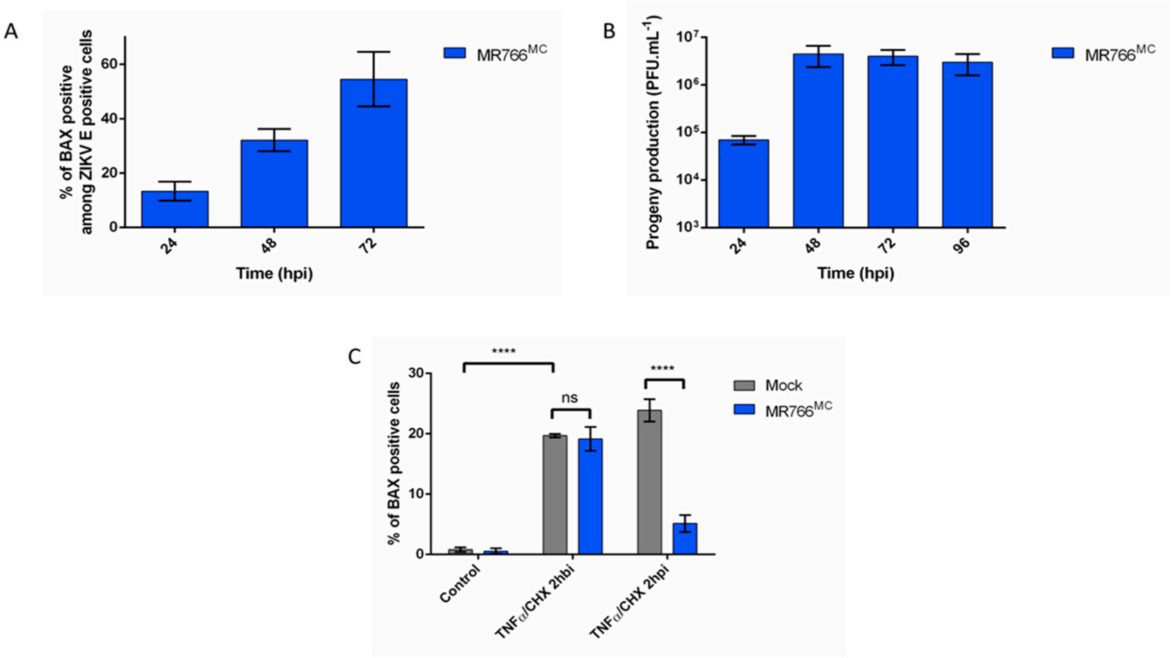

Figure 4. ZIKV-MR766 does not cause significant activation of apoptosis until late in infection and ZIKV-MR766 is able to control cell death. A549 cells were infected with MR766MC at MOI of 1 and during 96h. The percentage of A549 infected cells immunostained with anti-BAX antibody at 24, 48 and 72 hpi (**A**). The infectious virus was collected in supernatant of infected cells at 24, 48, 72 and 96 hpi for titration (PFU assay) (**B**). A549 cells were infected with MR766MC at MOI of 1 for 8 h and treated with TNFα/CHX 2 hours before infection (2hbi) or 2 hours post infection (2hpi). Percentage of A549 cells immunostained with anti-BAX antibody (**C**). Values represent the mean and standard deviation of three independent experiments. Data were analyzed by a one-way ANOVA test with post-hoc Tukey's test. (*****p* < 0,0001, ns: not significant).

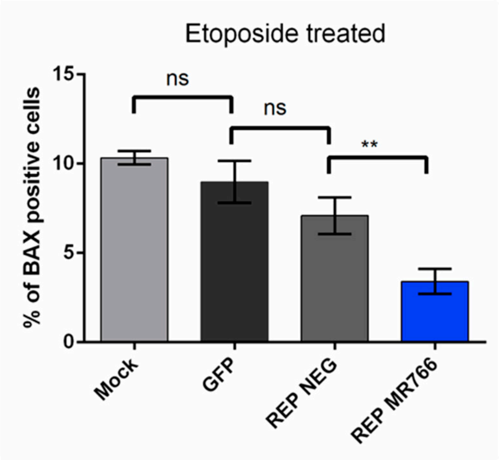

Figure 5. A549 cells transiently expressing a ZIKV-MR766 replicon are protected against cell death by apoptosis. A549 cells were transfected with amplicons of ISA methods to generate replicon for ZIKV-MR766 or with pEGFP-N1. 48h after transfection A549 cells were treated with Etoposide for 16 h. Percentage of A549 cells immunostained with BAX antibody were monitored. Values represent the mean and standard deviation of three independent experiments. Data were analyzed by a one-way ANOVA test with post-hoc Tukey's test (***p* < 0,01, ns: not significant).
